# Anxiety and Depression in Patients With Inflammatory Bowel Disease at King Fahad Specialist Hospital, Qassim Region

**DOI:** 10.7759/cureus.44895

**Published:** 2023-09-08

**Authors:** Khaled Alnafisah, Haifa N Alsaleem, Fai N Aldakheel, Areej b Alrashidi, Reema A Alayid, Hisham N Almuhayzi, Yazeed m Alrebdi

**Affiliations:** 1 Gastroenterology, King Fahad Specialist Hospital, Buraydah, SAU; 2 Medicine and Surgery, Qassim University, Buraydah, SAU; 3 Internal Medicine, King Fahad Specialist Hospital, Buraydah, SAU

**Keywords:** ulcerative colitis (uc), crohn’s disease, depression, anxiety, inflammatory bowel disease

## Abstract

Background

Inflammatory bowel disease (IBD) includes Crohn’s disease (CD) and ulcerative colitis (UC). It carries a risk of annual relapses and multiple intense flares, which require lifelong treatment and, sometimes, surgical interventions. This affects patients negatively in various aspects of their functioning, and they are left with an increased risk of disturbed quality of life and mental illnesses.

Aim

This study is carried out to describe the prevalence and risk factors of anxiety and depression symptoms among adult patients with IBD at King Fahad Specialist Hospital, Qassim, Saudi Arabia, and to examine the relationship between mental illness and disease activity.

Patient and methods

This cross-sectional descriptive study targets adult patients with IBD attending a gastroenterology clinic and medical day care unit. A self-administered questionnaire was given to patients with the help of an IBD nurse. The questionnaire includes the patient's demographic data (i.e., age, gender, etc.), symptoms and treatment patterns, the General Anxiety Disorder (GAD) questionnaire to measure anxiety, and the Patient Health Questionnaire (PHQ-9) to measure depression.

Results

Among the 179 IBD patients, 60.9% were males, and 40.8% were in the age group of 25-35 years. CD was the most prevalent IBD (73.2%). Perineal CD was detected in 45%. The prevalence of patients who were positive for anxiety symptoms was 17.3%, while the prevalence of depressive symptoms was 19.6%. The independent risk factor for anxiety was female gender, while the independent risk factor for depression was extraintestinal manifestation. The preventive factor for depression was treatment with IV medication.

Conclusion

Almost one-fifth of IBD patients were considered to have either anxiety or depression. Female gender was a risk factor for anxiety while extraintestinal manifestation of IBD was a risk factor for depression. Interestingly, treatment with IV medication was found to be the protective factor for depression. More investigations are warranted to give more insights regarding the prevalence and risk factors of psychological disorders among patients with IBD in our region.

## Introduction

Inflammatory bowel disease (IBD) is a chronic relapsing intestinal inflammation, including mainly Crohn's disease (CD) and ulcerative colitis (UC) [1]. It is a major health issue with a growing global prevalence. Research into the pathogenesis of IBD discovered that it results from various interactions of genetic, immune, and environmental factors [2]. The disease flare can be intense and severe, causing significant physical symptoms such as bloody diarrhea, abdominal pain, and fistulas [3]. Therefore, most cases require lifelong medical treatment, and many also require surgical intervention in the course of their disease [4]. This has a negative impact on the physical, psychological, familial, and social dimensions of those who are affected by it [5].

Having a chronic medical condition is associated with higher rates of, and is a risk factor for, psychiatric disorders, particularly anxiety and depression, compared to the general population [6,7]. Furthermore, IBD has an unpredictable course, with approximately 25-50% of patients relapsing annually [7]. Anxiety and depression independently are known to affect the quality of life and lead to functional impairment, even though they are still highly underdiagnosed health issues [8]. This, coupled with the profound effect of IBD on function as a result of systemic symptoms, surgery, and medication side effects [9], shows how significantly IBD affects the quality of life of IBD patients and the importance of detection of comorbid anxiety and depression [10,11]. The latter is important not only due to the high risk of co-occurrence but also due to the impact of anxiety and depression on the prognosis of IBD, as the presence of anxiety and depression leads to poor treatment compliance and higher morbidity and mortality [12-14]. Subsequently, it increases the burden on hospitals and prolongs hospital stays [12].

In this study, we aim to detect the prevalence of anxiety and depression in IBD patients who attend the outpatient gastroenterology clinic and medical day care unit at King Fahad Specialist Hospital in Buraydah, Qassim Region, Saudi Arabia. We also aim to detect factors that contribute to the increased risk of these two diseases co-occurring together.

## Materials and methods

This was a cross-sectional descriptive study conducted at the gastroenterology clinic and medical day care unit of King Fahad Specialist Hospital, Buraydah, Qassim region, Saudi Arabia, from March 2023 to June 2023. The study was approved by the Qassim Research Ethics Committee (approval number: 607/44/12287).

Sample size and sampling technique

Inclusion criteria were: Patients diagnosed with IBD, patients aged above 18 years and below 65 years, patients who consent to participate, and patients presenting to the gastroenterology clinic and medical day care unit. Patients who were not diagnosed with IBD, who were younger than 18 years and older than 65, and who did not consent to participate were excluded. A total of 179 IBD patients who attended the gastroenterology clinic and medical day care unit during the study period fulfilled the inclusion criteria and were included in the study.

Data collection

Data was collected from the IBD patients who participated in the study after obtaining their consent. This was done via a self-administered questionnaire in Arabic, consisting of 24 questions; six questions were on patient demographic data (file number, age, sex, type of IBD (UC or CD), time since diagnosis, history of surgery, smoking), 10 were about depression symptoms using validated Patient Health Questionnaire (PHQ-9), and eight were about anxiety using a validated General Anxiety Disorder (GAD) questionnaire.

The remaining data were collected from patients’ files such as the presence of perianal symptoms, disease extension and behavior, presence of extra-intestinal manifestations, oral medications, IV medication and type, subcutaneous medications, and anti-tumor necrosis factor (TNF) response. 

Questionnaire criteria

The anxiety symptoms were assessed by using the GAD-7. This is a seven-item questionnaire with a four-point Likert scale category ranging from "Not at all" coded with 0 to "Nearly every day" coded with 3. The GAD-7 score ranges from 0 to 21 points. A higher score indicates higher anxiety symptoms. The severity of anxiety was classified as minimal (score 0-4), mild (score 5-9), moderate (score 10-14), and severe (score 15-21). Finally, a score of 10 or higher indicates positive anxiety symptoms [15].

The depressive disorder was measured using the PHQ-9. This is a nine-item questionnaire with a four-point Likert scale category ranging from "Not at all" coded with 0 to "Nearly every day" coded with 3. The PHQ-9 score ranges between 0 to 27 points. A higher score suggests higher depressive symptoms. The severity of depression was considered minimal (score 1-4), mild (score 5-9), moderate (score 10-14), moderately severe (score 15-19), and severe (score 20-27). Finally, a score of 10 or higher was considered a positive depressive symptom [16].

Statistical analysis

Categorical data were described as frequency and proportion (%). Continuous data were computed and presented as mean and standard deviation. The association between anxiety and depression according to the patient's socio-demographic, symptoms, and treatment patterns was calculated using the Chi-square test. Significant findings were then placed in a multivariate regression estimate to determine the significant independent risk factors for anxiety and depression. A cutoff point of p<0.05 was taken to indicate significance. All statistical data were tabulated and analyzed using IBM SPSS Statistics for Windows, Version 26.0 (Released 2019; IBM Corp., Armonk, New York, United States).

## Results

A total of 179 patients with IBD were reviewed. As seen in Table 1, 40.8% were aged between 25 and 35 years, with males being dominant (60.9%). The most common type of IBD was CD (73.2%). Nearly half (49.2%) had been diagnosed with IBD less than five years prior to the study. Patients who had a previous history of surgery due to IBD were 30.7%. In addition, 14.5% were smokers. 

**Table 1:** Sociodemographic and clinical characteristics of participants (n=179)

Study variables	N (%)
Age group	
<25 years	60 (33.5%)
25 – 35 years	73 (40.8%)
36 – 45 years	26 (14.5%)
>45 years	20 (11.2%)
Gender	
Male	109 (60.9%)
Female	70 (39.1%)
Type of inflammatory bowel disease?	
Crohn's Disease	131 (73.2%)
Ulcerative Colitis	48 (26.8%)
Time since diagnosis	
<5 years	88 (49.2%)
5 – 10 years	64 (35.8%)
>10 years	27 (15.1%)
Previous surgery related to inflammatory bowel disease?	
Yes	55 (30.7%)
No	124 (69.3%)
Smoking	
Yes	26 (14.5%)
No	153 (85.5%)

The prevalence of patients with perineal CD was 45% (Table 2), and the most common behavior was stricturing (66.4%), while Ileocolonic was the most common CD extension (38%). The most common UC disease extension was pancolitis (52.1%). Skin was the most common extraintestinal manifestation (22.3%). The most preferred oral medication was azathioprine (49.7%), and infliximab was the most preferred IV medication. Also, adalimumab was the most frequently used subcutaneous medication (14.5%). Additionally, 20.1% had a history of anti-TNF failure.

**Table 2:** Symptoms and treatment patterns (n=179) ^†^Some patients have one or more Crohn's Disease behaviors.

Variables	N (%)
Perineal Disease (Crohn's Disease) (n=131)	
Yes	59 (45.0%)
No	72 (55.0%)
Behavior (Crohn's Disease) (n=131) ^†^	
B1: non-stricturing, non-penetrating	18 (13.7%)
B2: stricturing	87 (66.4%)
B3: penetrating	68 (51.9%)
Disease extension (Crohn's Disease)	
L1: Ileal	50 (27.9%)
L2: Colonic	12 (06.7%)
L3: Ileocolonic	68 (38.0%)
L4: Isolated upper disease	01 (0.60%)
Disease extension (Ulcerative colitis) (n=48)	
E1: Proctitis	01 (02.1%)
E2: Left-sided	23 (47.9%)
E3: Pancolitis	25 (52.1%)
Extraintestinal Manifestations	
None	91 (50.7%)
Eye	14 (07.8%)
Skin	40 (22.3%)
Joints	33 (18.4%)
PSC	01 (0.60%)
Oral Medication	
None	56 (31.3%)
Azathioprine	89 (49.7%)
Mesalamine	26 (14.5%)
Azathioprine and Mesalamine	07 (03.9%)
Upadacitinib	01 (0.60%)
IV medication	
None	65 (36.3%)
Infliximab	108 (60.3%)
Vedolizumab	06 (03.4%)
Subcutaneous Medication	
None	131 (73.2%)
Adalimumab	26 (14.5%)
Ustekinumab	22 (12.3%)
History of Anti-TNF Failure	
Yes	36 (20.1%)
No	143 (79.9%)

The assessment of anxiety and depression has been described in Table 3. It can be observed that the mean anxiety score was 5.16 (SD 4.84), with minimal, mild, moderate, and severe levels constituting 54.7%, 27.9%, 10.6%, and 6.7%, respectively. Accordingly, we found that the prevalence of patients who were considered to have positive anxiety symptoms was 17.3%. Regarding depression, the overall mean depression score was 5.96 (SD 5.28), with minimal, mild, moderate, moderately severe, and severe levels found among 46.9%, 33.5%, 10.6%, 6.1%, and 2.8%, respectively. The prevalence of patients who were considered as having positive depression was 19.6%, while 80.4% had no symptoms of depression.

**Table 3:** Prevalence of anxiety using general anxiety disorder (GAD-7) questionnaire and depression using patient health questionnaire (PHQ-9)

Variables	N (%)
Anxiety score (mean ± SD)	5.16 ± 4.84
Severity of anxiety	
Minimal (score 0 – 4)	98 (54.7%)
Mild (score 5 – 9)	50 (27.9%)
Moderate (score 10 – 14)	19 (10.6%)
Severe (score 15 – 21)	12 (06.7%)
Symptoms of anxiety	
Negative (score <10)	148 (82.7%)
Positive (score ≥10)	31 (17.3%)
Depression score (mean ± SD)	5.96 ± 5.28
Severity of depression	
Minimal (score 0 – 4)	84 (46.9%)
Mild (score 5 – 9)	60 (33.5%)
Moderate (score 10 – 14)	19 (10.6%)
Moderately severe (score 15 – 19)	11 (06.1%)
Severe (score 20 – 27)	05 (02.8%)
Symptoms of depression	
Negative (score <10)	144 (80.4%)
Positive (score ≥10)	35 (19.6%)

When measuring the relationship between anxiety symptoms with the patients' sociodemographic and clinical characteristics (Table 4), it was found that the prevalence of patients with anxiety symptoms was significantly more common in the female gender (p=0.005), those with longer duration of IBD (p=0.038), and those who were taking subcutaneous medication (p=0.046).

**Table 4:** Relationship between anxiety and sociodemographic and clinical characteristics ^†^ Variable with multiple response answers; ^§^ P-value has been calculated using Chi-square test; ** Significant at p<0.05 level.

Factor	Anxiety symptoms		P-value ^§^
	Positive, N (%) (n=31)	Negative, N (%) (n=148)	
Age group			
<25 years	13 (41.9%)	47 (31.8%)	0.079
25 – 35 years	15 (48.4%)	58 (39.2%)	
>45 years	03 (09.7%)	43 (29.1%)	
Gender			
Male	12 (38.7%)	97 (65.5%)	0.005 **
Female	19 (61.3%)	51 (34.5%)	
Type of inflammatory bowel disease			
Crohn’s disease	22 (71.0%)	109 (73.6%)	0.759
Ulcerative colitis	09 (29.0%)	39 (26.4%)	
Time since diagnosis			
<5 years	10 (32.3%)	78 (52.7%)	0.038 **
≥5 years	21 (67.7%)	70 (47.3%)	
Previous surgery related to inflammatory bowel disease			
Yes	10 (32.3%)	45 (30.4%)	0.839
No	21 (67.7%)	103 (69.6%)	
Smoking			
Yes	06 (19.4%)	20 (13.5%)	0.401
No	25 (80.6%)	128 (86.5%)	
Extraintestinal manifestation			
Yes	17 (54.8%)	55 (37.2%)	0.068
No	14 (45.2%)	93 (62.8%)	
Oral medication			
Yes	19 (61.3%)	104 (70.3%)	0.327
No	12 (38.7%)	44 (29.7%)	
IV medication			
Yes	16 (51.6%)	98 (66.2%)	0.124
No	15 (48.4%)	50 (33.8%)	
Subcutaneous medication			
Yes	13 (41.9%)	36 (24.3%)	0.046 **
No	18 (58.1%)	112 (75.7%)	
Anti-TNF failure			
Yes	10 (32.3%)	26 (17.6%)	0.064
No	21 (67.7%)	122 (82.4%)	

When measuring the relationship between depressive symptoms and the sociodemographic and clinical characteristics of the patients (Table 5), it was observed that the prevalence of patients with depressive symptoms was significantly more common among those with extraintestinal manifestation (p=0.002), while the prevalence was significantly less among those who received IV medication (p=0.014).

**Table 5:** Relationship between depression according to the patient's socio-demographic and clinical characteristics ^†^ Variable with multiple response answers; ^§^ P-value has been calculated using Chi-square test; ** Significant at p<0.05 level. TNF: tumor necrosis factor

Factor	Depressive symptoms		P-value ^§^
	Positive, N (%) (n=35)	Negative, N (%) (n=144)	
Age group			
<25 years	15 (42.9%)	45 (31.3%)	0.184
25 – 35 years	15 (42.9%)	58 (40.3%)	
>45 years	05 (14.3%)	41 (28.5%)	
Gender			
Male	18 (51.4%)	91 (63.2%)	0.201
Female	17 (48.6%)	53 (36.8%)	
Type of inflammatory bowel disease			
Crohn’s disease	25 (71.4%)	106 (73.6%)	0.794
Ulcerative colitis	10 (28.6%)	38 (26.4%)	
Time since diagnosis			
<5 years	14 (40.0%)	74 (51.4%)	0.227
≥5 years	21 (60.0%)	70 (48.6%)	
Previous surgery related to inflammatory bowel disease			
Yes	11 (31.4%)	44 (30.6%)	0.920
No	24 (68.6%)	100 (69.4%)	
Smoking			
Yes	06 (17.1%)	20 (13.9%)	0.624
No	29 (82.9%)	124 (86.1%)	
Extraintestinal manifestation			
Yes	22 (62.9%)	50 (34.7%)	0.002 **
No	13 (37.1%)	94 (65.3%)	
Oral medication			
Yes	21 (60.0%)	102 (70.8%)	0.215
No	14 (40.0%)	42 (29.2%)	
IV medication			
Yes	16 (45.7%)	98 (68.1%)	0.014 **
No	19 (54.3%)	46 (31.9%)	
Subcutaneous medication			
Yes	14 (40.0%)	35 (24.3%)	0.062
No	21 (60.0%)	109 (75.7%)	
Anti-TNF failure			
Yes	10 (28.6%)	26 (18.1%)	0.164
No	25 (71.4%)	118 (81.9%)	

When conducting a multivariate regression model (Table 6), it was revealed that the female gender was the significant independent risk factor for anxiety. This further suggests that compared to male patients, female patients were predicted to have at least three times increased risk of having anxiety (adjusted odds ratio (AOR)=3.069; 95%CI=1.358-6.935; p=0.007). On the other hand, extraintestinal manifestation was the significant independent risk factor for depression, while treatment with IV medication was the independent preventive factor. This further indicates that patients with extraintestinal manifestation were 2.9 times more likely to have depressive symptoms (AOR=2.918; 95%CI=1.340-6.357; p=0.007). In contrast, patients who received IV medication were predicted to decrease the risk of depression by at least 56% (AOR=0.443; 95%CI=0.205-0.959; p=0.039).

**Table 6:** Multivariate regression analysis to determine the significant independent risk factors for anxiety and depression ^**^ Significant at p<0.05 level.

Anxiety	Adjusted OR	95% CI	P-value
Gender			
Male	Ref		
Female	3.069	1.358 – 6.935	0.007 **
Time since diagnosis			
<5 years	Ref		
≥5 years	1.916	0.788 – 4.659	0.151
Subcutaneous medication			
No	Ref		
Yes	1.873	0.774 – 4.536	0.164
Depression			
Extraintestinal manifestation			
No	Ref		
Yes	2.918	1.340 – 6.357	0.007 **
IV medication			
No	Ref		
Yes	0.443	0.205 – 0.959	0.039 **

## Discussion

This study was carried out to investigate the prevalence of and risk factors for the symptoms of anxiety and depression, as well as to examine if there is an existing relationship between mental illness and disease activity among adult patients with IBD. This study validates a high prevalence of anxiety among IBD patients. According to the GAD-7 criteria, the prevalence of patients with anxiety symptoms was 17.3%, with mild symptoms constituting 27.9%, moderate levels were 10.6%, and the rest were severe (6.7%). The overall mean anxiety score was 5.16 (SD 4.84). with an anxiety prevalence of 21.2%. Similarly, using the Depression, Anxiety, and Stress Scale (DASS-21), Smolović et al. found that the prevalence of anxiety among IBD patients was 14.9% [16]. The overall pool prevalence of anxiety among IBD patients was 32.1%. 

Results of our study revealed that anxiety symptoms were highly prevalent among female patients, increasing years of IBD, and patients taking subcutaneous medications. However, in our multivariate regression model, only the female gender remained significant and was determined as the significant independent risk factor for anxiety. This mirrored the study by Byrne et al. done in Canada [11], as they too found that females were also more likely to exhibit anxiety than males. This has been concurred by a literature review done in Italy [17], reporting that most studies validated that women with IBD were more likely than men to develop psychiatric disorders, yielding up to 65% of women with IBD. 

A study done by Evertsz et al. reported that CD was a risk factor for anxiety. The finding suggests that compared to control, anxiety new onset hazard ratio (HR) for the diagnosis of CD and UC were 1.63 and 6.60, respectively. However, this has not been the case in our study, as we found no significant relationship between the type of IBD and the symptoms of anxiety (p=0.759).

Another determinant of psychological disorders among the IBD population was depression. In our study, the prevalence of depression among patients with IBD was 19.6% (PHQ-9 mean score 5.96; SD 5.28). Accordingly, mild, moderate, moderately severe, and severe depression were found in 33.5%, 10.6%, 6.1%, and 2.8%, respectively. In a study in Jeddah, based on HADS-D criteria, 30.1% of the patients had borderline HADS-D scores [13], while according to a study done in Montenegro, 19.1% of the patients had been diagnosed with moderate depression, and 20.2% were stressed [16]. The lowest prevalence of depression among IBD patients has been documented in France at 11% [14]. 

Data in our study indicates that extraintestinal manifestation was the lone significant risk factor for depression, whereas treatment with IV medication was likely to be the protective factor against depression. Other sociodemographic and clinical variables were irrelevant, including age, gender, IBD duration, previous IBD-related surgery, smoking, subcutaneous medication, and anti-TNF failure (p>0.05). These findings contradicted the results of Nahon et al., who indicated that age, unemployed status, socioeconomic levels, flares, and disability were the factors associated with depression [14]. Similarly, Cheema et al. reported that younger age, lack of access to an IBD nurse, and lack of information on reducing infection risk were all independently associated with anxiety/depression [18]. Incidentally, Gao et al. revealed that CD-related surgery and CD activity index (CDAI) were identified as independent risk factors for symptoms of anxiety/depression in CD, while corticosteroid use was determined as an independent risk factor for anxiety/depression symptoms at UC [19]. However, our study found no significant relationship between the level of depression in terms of CD and UC, which contradicted previous reports. 

Moreover, a study carried out among Canadian IBD patients reported that 30.1% suffered from anxiety and depression [11]. Likewise, a study done in Montenegro found that nearly one-third of the IBD patients were suffering from at least one of the examined mental disorders during the coronavirus disease 2019 (COVID-19) pandemic [16]. In our study, however, approximately 14% of the patients had symptoms of both anxiety and depression. These findings indicate the need for psychiatric treatment referral. Screening for and treating psychiatric symptoms should become integral to IBD medical care.

Limitations

The current study had some limitations including the cross-sectional design of the study, which makes it difficult to find a causal relation between anxiety, depression, and inflammatory bowel disease. As anxiety and depression can be affected by various factors, investigating each one was not possible within this study. Also, only the patients who attended the gastroenterology clinic and medical day care unit at King Fahad Specialist Hospital between March and June 2023 were included.

## Conclusions

Nearly one-fifth of IBD patients had either anxiety or depression. Females were more likely to exhibit anxiety but not depression. Extraintestinal manifestation increases the risk of depression, while treatment with IV medication decreases its chances. Constant monitoring among IBD patients is necessary, particularly among those who are showing signs of mental illness. Furthermore, women with IBD require regular monitoring for anxiety. Increased awareness among clinicians is vital, as these disorders may affect response to treatment and quality of life among this group of patients.
